# Body composition and bone mineral density in athletes with a physical impairment

**DOI:** 10.7717/peerj.11296

**Published:** 2021-05-10

**Authors:** Valentina Cavedon, Marco Sandri, Ilaria Peluso, Carlo Zancanaro, Chiara Milanese

**Affiliations:** 1 Department of Neurosciences, Biomedicine and Movement Sciences, University of Verona, Verona, Italy; 2 Council for Agricultural Research and Economics (CREA-AN), Research Centre for Food and Nutrition, Rome, Italy

**Keywords:** DXA, Spinal cord injury, Lower limb amputation, Fat-mass to lean-mass ratio, Bone mineral density, Percentage fat mass, Body composition

## Abstract

**Background:**

The impact of the type and the severity of disability on whole-body and regional body composition (BC), and bone mineral density (BMD) must be considered for dietary advice in athletes with a physical impairment (PI). This study aimed to investigate the impact of the type and the severity of disability on BC, the pattern of distribution of fat mass at the regional level, and BMD in athletes with a PI.

**Methods:**

Forty-two male athletes with spinal cord injury (SCI, *n* = 24; age = 40.04 ± 9.95 years, Body Mass Index [BMI] = 23.07 ± 4.01 kg/m^2^) or unilateral lower limb amputation (AMP, *n* = 18; age = 34.39 ± 9.19 years, BMI = 22.81 ± 2.63 kg/m^2^) underwent a Dual-Energy X-Ray Absorptiometry scan. Each athlete with a PI was matched by age with an able-bodied athlete (AB, *n* = 42; age = 37.81 ± 10.31 years, BMI = 23.94 ± 1.8 kg/m^2^).

**Results:**

One-Way Analysis of Variance showed significant differences between the SCI, AMP and AB groups for percentage fat mass (%FM) (*P* < 0.001, eta squared = 0.440). Post-hoc analysis with Bonferroni’s correction showed that athletes with SCI had significantly higher %FM vs. the AMP and AB groups (25.45 ± 5.99%, 21.45 ± 4.21% and 16.69 ± 2.56%, respectively; *P* = 0.008 vs. AMP and *P* < 0.001 vs. AB). The %FM was also significantly higher in the AMP vs. the AB group (*P* < 0.001). Whole-body BMD was negatively affected in SCI athletes, with about half of them showing osteopenia or osteoporosis. In fact, the mean BMD and T-score values in the SCI group (1.07 ± 0.09 g/cm^2^ and −1.25 ± 0.85, respectively) were significantly lower in comparison with the AB group (*P* = 0.001 for both) as well as the AMP group (*P* = 0.008 for both). The type of disability affected BC and BMD in the trunk, android, gynoid and leg regions in SCI athletes and the impaired leg only in AMP athletes.

**Conclusions:**

In conclusion, the type of disability and, partly, the severity of PI impact on BC and BMD in athletes with a PI. Nutritionists, sports medicine doctors, clinicians, coaches and physical conditioners should consider athletes with SCI or AMP separately. Athletes with a PI would benefit from specific nutrition and training programs taking into account the type of their disability.

## Introduction

Increased fat mass (FM) and/or loss of lean mass (LM) leading to an increase in fat-to-lean mass ratio (FM/LM) ratio often takes place in people with a physical impairment (e.g., spinal cord injury caused by paralysis (SCI) or lower limb amputation (AMP)) (Dionyssiotis et al., 2008; Sherk, Bemben & Bemben, 2010). In these individuals exercise training improves body composition (Liu, Wang & Niebauer, 2021) and its accurate measure is fundamental for a personalized nutrition and training programs, in particular in athletes with a physical impairment (Bernardi et al., 2020).

Research on this athletic population has highlighted that the above-described adverse changes in whole-body and regional body composition are prevented/mitigated by the regular practice of an adapted sport (Inukai et al., 2006; Gorla et al., 2016; Cavedon, Zancanaro & Milanese, 2018; Cavedon, Zancanaro & Milanese, 2020). Of the available literature, several studies on body composition focused either on athletes with SCI (Inukai et al., 2006; Miyahara et al., 2008; Mojtahedi et al., 2008; Mojtahedi, Valentine & Evans, 2009; Willems et al., 2015; Gorla et al., 2016; Goosey-Tolfrey et al., 2016; Flueck, 2020) or on mixed samples of athletes with different types and/or degrees of severity of their physical impairment including, for example, chronic arthritis, spinal cord injury, dystrophic dysplasia, multiple sclerosis and lower limb nerve damage (Sutton et al., 2009; Willems et al., 2015; Goosey-Tolfrey et al., 2016; Keil et al., 2016; Cavedon, Zancanaro & Milanese, 2020).

The results of the above-reported studies, however, lack information about some important aspects of body composition as well as the bone status of athletes. For example, the impact of the type and the severity of the disability in athletes with a physical impairment remains essentially unknown with regards to whole-body and regional body composition, bone mineral density (BMD) and the regional distribution of FM. Moreover, information about athletes with AMP as a separate group as well as the frequency of obesity and osteopenia/osteoporosis has never been reported in studies dealing with athletes with a locomotor impairment.

Another neglected issue in the investigation of body composition in athletes with a physical impairment is the amount of FM in the android and gynoid regions as well as the whole-body and regional FM/LM ratio. The assessment of FM accumulation in the android region is important from a health perspective as the central accumulation of FM is a well-recognized risk factor for metabolic and cardiovascular diseases (Goldstein et al., 2017; Stillman & Williams, 2019). Furthermore, it has been recently pointed out the relevance of the resting energy expenditure to LM ratio to determine the nutrient intake needs for athletes with SCI (Broad et al., 2020), whereas Eckard et al. (2015) did not find changes in resting metabolism or walking energy expenditure during the first year following traumatic amputation, despite the body composition changes.

In order to fill some of these important gaps in the scientific literature, this study assessed whole-body and regional body composition and BMD in athletes with a physical impairment, distinguishing athletes according to the type and the severity of disability. The scientific data provided in this study would be useful for nutritionist, physical conditioners, coaches and sports medicine doctors to personalize nutrition and training programs. The aim of this study was twofold. First, to investigate the impact of the type of disability on whole-body and regional body composition, the pattern of distribution of FM at the regional level and BMD in athletes with a physical impairment. Second, to explore the impact of the severity of the disability on whole-body and regional body composition in athletes with SCI according to the level of injury (i.e., injury at the cervical level or injury at the thoracic/lumbar level) and in athletes with AMP according to the level of amputation (i.e., amputation above the knee or amputation below the knee). According to the literature (Willems et al., 2015; Sherk, Bemben & Bemben, 2008, 2010), it is hypothesized that, in athletes with a physical impairment the alterations in BC and in bone parameters as well as the regional distribution of body tissues are affected by the type and, possibly, by the severity of the physical impairment.

## Materials & methods

### Participants

The study was conducted in accordance with the Declaration of Helsinki, and the protocol was approved by the Institutional Review Board of the University of Verona (Protocol number: 18198, 05/04/2013). All participants were volunteers and signed an informed consent form.

Forty-two Caucasian male athletes with a physical impairment were enrolled in this cross-sectional study. The adapted sports practiced by athletes were para table tennis (*n* = 1), handbike (*n* = 10), wheelchair rugby (*n* = 8), wheelchair basketball (*n* = 9), paratriathlon (*n* = 1) and amputee soccer (*n* = 13). Inclusion criteria were the participation in an adapted sport at a competitive level for at least two years and, regular training (i.e., at most one break period from sport activity not greater than 3 months per competitive season). According to the type of physical impairment, athletes were divided into two groups: athletes with SCI (*n* = 24) and athletes with AMP (*n* = 18). The SCI group comprised athletes with SCI at the cervical level (*n* = 12; TETRA), and at the thoracic or lumbar level (PARA, *n* = 12). Disability in the AMP group comprised amputation through the hip or transfemoral amputation (AKA, *n* = 11), and amputation through the knee or transtibial amputation (BKA, *n* = 7).

Each athlete with a physical impairment was matched with an able-bodied (AB) Caucasian male athlete of the same age. Able-bodied athletes in the control group (AB athletes, *n* = 42) were randomly selected with an age-stratified random sampling method from a larger group composed of 242 non-professional athletes who were competing in different sport activities (e.g., soccer, basketball, rugby, volleyball, track and field, tennis, cycling, long-distance running).

### Testing procedures

Data were collected as previously described in Cavedon and colleagues (Cavedon et al., 2020). Specifically, testing took place in the late morning/early afternoon, after a 3–4 h fast. All participants were asked not to undertake any strenuous physical activity the day before each measurement session and they were also required not to undertake any exercising on the day of the measurements.

A face-to-face interview was conducted to confirm the participants’ eligibility criteria and to collect the following information: date of birth, type and severity of disability, duration of injury (DOI), sport practiced, years of sport experience and amount of weekly training.

#### Anthropometric assessment

Body mass and stature are required by the DXA software to enable scanning and were assessed as follows. For athletes who were able to stand up, body mass was assessed to the nearest 0.1 kg with an electronic scale (Tanita electronic scale BWB-800 MA) and stature was measured to the nearest 0.5 cm with a Harpenden stadiometer (Holtain Ltd., Crymych, Pembs. UK) according to conventional criteria and measuring procedures (Lohman, Roche & Martorell, 1988). For athletes who were wheelchair users, and thus unable to stand up, body mass and stature were self-reported. For all participants the Body Mass Index (BMI) was calculated as body mass (kg)/height^2^ (m^2^).

#### Body composition and BMD assessment

Body composition and BMD were assessed by means of DXA using a total body scanner (QDR Horizon, Hologic MA, USA; fan-beam technology, software for Windows XP version 13.6.05), according to Cavedon and colleagues (Cavedon et al., 2020). Specifically, athletes were asked to void their bladder and to remove all metal, jewelry or reflective material, including prostheses where possible. During the DXA scanning athletes wore only underwear. Athletes undertook DXA scanning according to “The Best Practice Protocol for the assessment of whole-body body composition by DXA” (Nana et al., 2015). Positioning aids to support the impaired lower limb of athletes in the AMP group were employed and special strapping was applied around athlete’s residual ankle to ensure there was no movement during the scan.

No movement artifacts were detected in scans and, accordingly, all scans were used in the analysis. Analysis of scans was performed by the same trained operator. The operator localized the specific anatomical landmarks to differentiate the standard regions of interests (trunk, arms [right and left], legs [right and left]). The android region was defined with a distal limit placed on top of the iliac crests and a proximal limit set at 20% of the distance from the top of the iliac crest to the base of the skull. The total height of the gynoid region was twice the height of the android region and was defined with a proximal limit positioned below the pelvis line by 1.5 times the height of the android region.

For the purpose of this study, the left and the right arm were considered one region (Arms) while the left and the right leg were considered separately. In the AMP group, the non-impaired leg was considered as the “right” leg while the impaired leg was considered as the “left”.

For a more detailed analysis, in the AMP group only the thigh and lower leg regions of both the impaired and non-impaired legs were computed according to Hart and colleagues (Hart et al., 2015). The thigh region was delineated by a proximal boundary formed by an oblique line passing through the femoral neck to a distal boundary formed by the horizontal line passing through the knee axis, noted as the space between the femoral and tibial condyles. The proximal boundary of the lower leg region was a horizontal line passing through the knee axis as described above, while the distal boundary was a horizontal line spanning beneath the medial and lateral malleoli.

#### Outcomes

The following body composition variables at the whole-body as well as regional level were considered: total mass, lean mass (LM), fat mass (FM), percentage FM (%FM), fat-to-lean mass ratio (FM/LM), bone mineral content (BMC), and bone mineral density (BMD). For the android and gynoid regions only FM and %FM were included in analysis.

Definition of obesity was having a %FM ≥ 25% (Romero-Corral et al., 2008). To interpret the BMD values and to define the ranges of osteopenia and osteoporosis, the T-scores were computed as the difference between the whole-body BMD of each athlete and the mean whole-body BMD of a reference population aged 30 years old. The reference population was composed by healthy white adult males from NAHNES database (Kelly, Wilson & Heymsfield, 2009). For one athlete aged 17, the reference population was composed of healthy Caucasian pediatric males from NAHNES database (Kelly, Wilson & Heymsfield, 2009). Osteopenia was defined as a T-score of −1 (i.e., one standard deviation below the mean of the reference population) and osteoporosis was defined as a T-score of −2.5 (i.e., 2.5 standard deviation below the mean of the reference population) (Kelly, Wilson & Heymsfield, 2009).

Due to the differences in body mass among the subjects of this study, which is associated with the absence of one or more body segments in AMP, only relative variables (i.e., %FM, FM/LM ratio and BMD) were analyzed when comparing athletes with a physical impairment with each other or with AB athletes. When investigating the impact of the severity of the disability in SCI and AMP groups, both the absolute and relative variables (i.e., total mass, LM, FM, %FM, FM/LM BMC and BMD) were considered in the analysis.

### Statistical analysis

Descriptive statistics (mean and standard deviation) were computed for all variables. Normality of data was assessed using the Kolmogorov-Sm–irnov test and, when necessary, data were transformed using the method described by Box & Cox (1964). The Levene’s test was applied to check homogeneity of variances.

The two-tailed Student *t*-test for independent samples and one-way Analysis of Variance (ANOVA) were carried out when comparing means between two and three groups, respectively. After one-way ANOVA, a post-hoc analysis with Bonferroni’s correction for multiple comparison was performed. Two-tailed paired sample *t*-test was also conducted to explore the differences between the whole-body %FM and the %FM assessed in the trunk, arms, both legs regions.

Eta squared (η^2^) was used to calculate the effect size in the Student *t*-test for independent samples and in the ANOVA, while Cohen’s d (d) was used to calculate the effect size in the paired sample *t*-test. According to Cohen (1988), effect size values were interpreted as small (η^2^ = 0.01 and d = 0.2), medium (η^2^ = 0.06 and d = 0.5), and large (η^2^ = 0.14 and d = 0.8).

All analysis was performed with SPSS v. 16.0 (IBM Corp., Armonk, NY, USA). Statistical significance was set at *P* ≤ 0.05.

## Results

### Characteristics of the athletes

The characteristics of the SCI and AMP groups as well as their relative sub-groups (TETRA and PARA; AKA and BKA, respectively) are summarized in Table 1. The mean age and BMI of athletes in the AB group was 37.81 ± 10.31 years and 23.94 ± 1.8 kg/m^2^, respectively.

**Table 1:** Characteristics of the SCI and AMP groups and their relative sub-groups.

	SCI (*n* = 24)		TETRA (*n* = 12)		PARA (*n* = 12)		AMP (*n* = 18)		AKA (*n* = 11)		ABK (*n* = 7)	
	Mean	SD	Mean	SD	Mean	SD	Mean	SD	Mean	SD	Mean	SD
Age (y)	40.04	9.95	36.75	10.22	43.33	8.90	34.39	9.19	34.91	10.22	33.57	7.98
BMI (kg/m^2^)	23.07	4.01	22.94	4.54	23.20	3.59	22.48	2.06	21.72	2.11	23.68	1.39
DOI (y)	15.75	8.58	12.83	6.66	18.67	9.55	12.00	9.49	11.45	8.86	12.86	11.08
Experience (y)	9.92	6.86	7.33	4.79	12.50	7.80	6.89	7.10	6.82	8.06	7.00	5.89
Training (h/w)	6.08	2.45	5.83	2.21	6.33	2.74	4.92	1.73	4.73	1.74	5.21	1.82

**Note:**

SCI, athletes with spinal cord injury; TETRA, athletes with SCI at the cervical level; PARA, athletes with SCI at the thoracic/lumbar level; AMP, athletes with lower limb amputation; AKA, athletes with above-knee amputation; BKA, athletes with below-knee amputation or with amputation through the knee; y, years; BMI, Body Mass Index; DOI, duration of injury; Training, amount of training; h/w, hours per week.

No significant differences were found between the SCI and the AMP groups in age, DOI, sport experience and amount of training. Similarly, no significant differences were found in age, DOI, sport experience and amount of training between the TETRA and PARA groups as well as AKA and BKA groups. One-way ANOVA also showed no significant differences between the SCI, AMP and AB groups in age (F = 1.653, *P* = 0.198; η^2^ = 0.04). The two-tailed Student *t*-test for independent samples showed a statistically significant difference in BMI between the AKA and BKA groups (t = −2.175, *P* = 0.045, η^2^ = 0.23).

### Impact of the type of disability on whole-body and regional body composition and BMD in athletes with a physical impairment

#### Whole-body analysis

**Figure 1:**
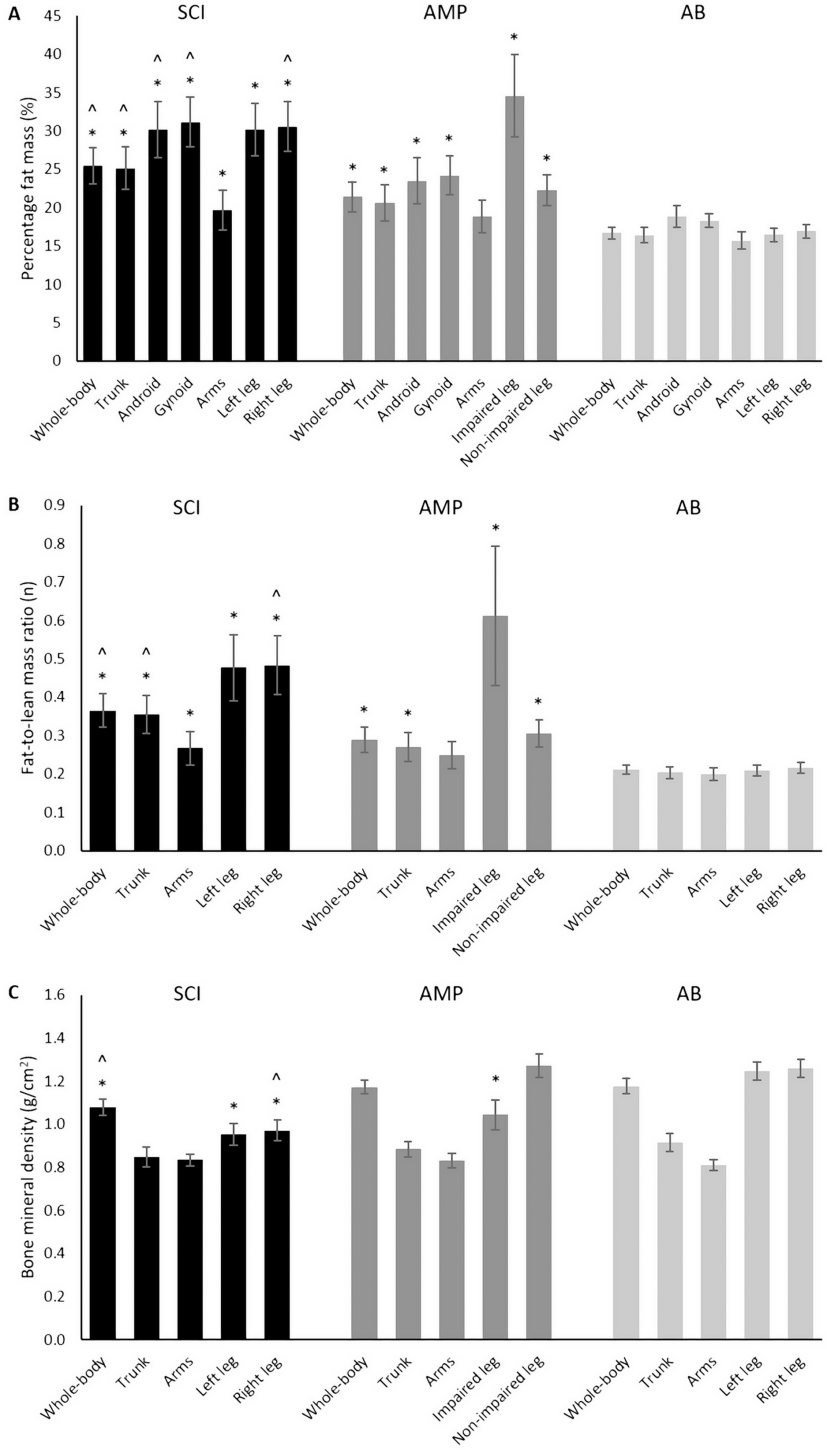
Percentage fat mass (A), fat-to-lean mass ratio (B) and bone mineral density (C) assessed in the SCI, AMP and AB groups. SCI, athletes with spinal cord injury; AMP, athletes with unilateral lower limb amputation; AB, able-bodied athletes. Data are mean with Confidence Intervals. *, significantly different from the AB group; ^, significantly different from the AMP group.

**Figure 2:**
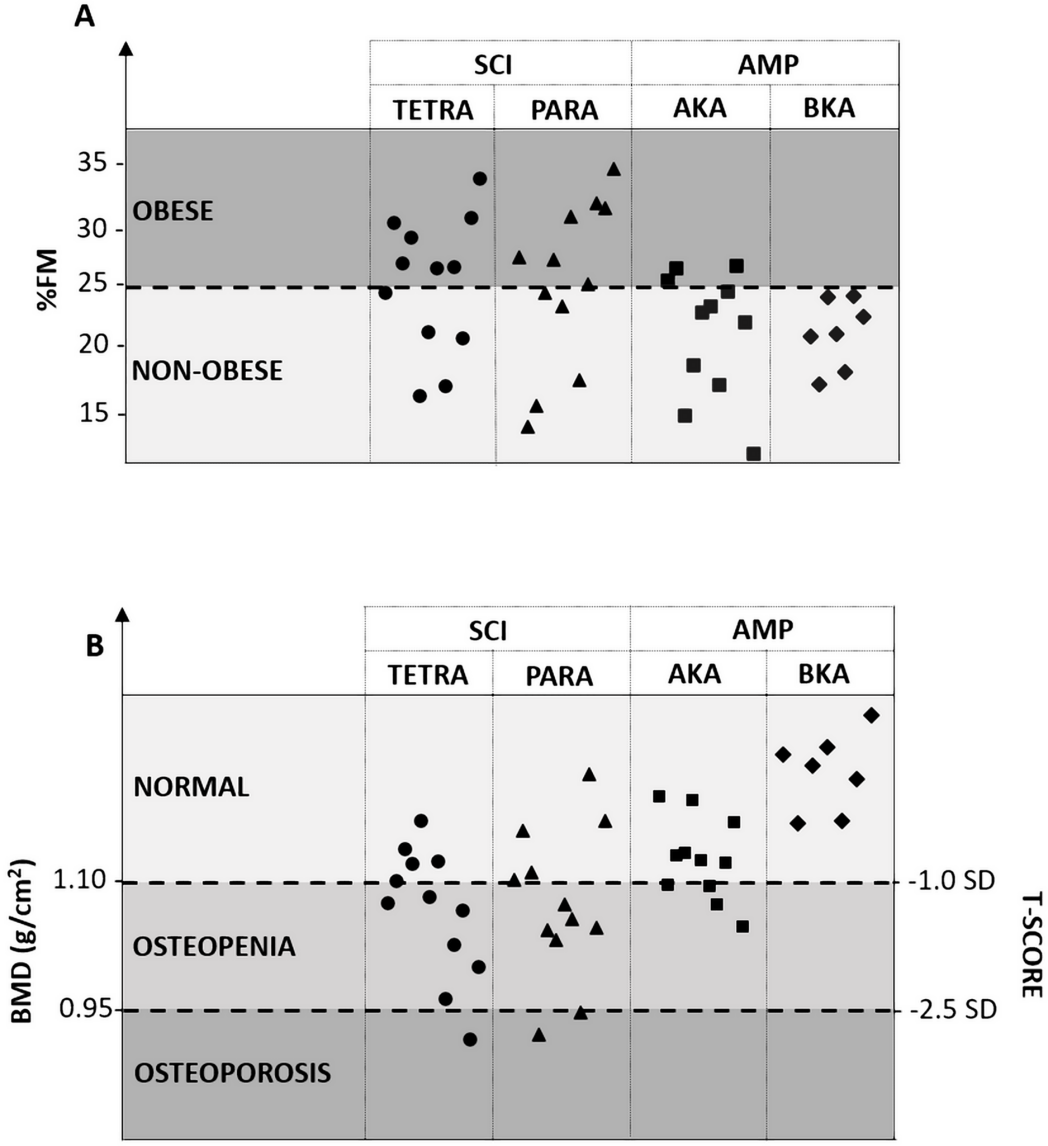
Presence of obesity (A) and/or osteopenia/osteoporosis (B) in athletes with a physical impairment according to the type and the severity of their disability. SCI, spinal cord injury; TETRA, athletes with spinal cord injury at the cervical level; PARA, athletes with spinal cord injury at the thoracic/lumbar level; AMP, unilateral lower limb amputation; AKA, athletes with unilateral lower limb amputation above the knee; BKA, athletes with unilateral lower limb amputation below (or through) the knee; %FM, percentage fat mass; BMD, bone mineral density; SD, standard deviation.

One-way ANOVA revealed significant differences between the SCI, AMP, and AB groups for %FM (F = 31.848, *P* < 0.001, η^2^ = 0.440). Post-hoc analysis showed that both the SCI and AMP groups had significantly higher %FM in comparison with the AB group (*P* < 0.001 for both; Fig. 1A). The SCI group had significantly higher %FM vs. the AMP group (*P* = 0.008; Fig. 1A). Based on the %FM threshold of 25%, 58.3% of athletes with SCI and 16.7% of athletes with AMP were obese (Fig. 2A).

Significant differences between the SCI, AMP, and AB groups were also found for FM/LM ratio (F = 35.446, *P* < 0.001, η^2^ = 0.467). Post-hoc analysis showed that both the SCI and AMP groups had significantly higher FM/LM ratio in comparison with the AB group (*P* < 0.001 for both; Fig. 1B). The SCI group had also significantly higher FM/LM versus the AMP group (*P* = 0.003; Fig. 1B).

One-way ANOVA revealed significant differences between the SCI, AMP, and AB groups for both BMD (F = 8.434, *P* = 0.001, η^2^ = 0.172) and T-score (F = 8.466, *P* < 0.001, η^2^ = 0.173). Post-hoc analysis showed that in the SCI group both BMD and T-score were significantly lower versus the AB group (*P* = 0.001 for both; Fig. 1C), whereas the AMP and AB groups had similar values for both variables (*P* > 0.05 for both; Fig. 1C). The SCI group had significantly lower BMD (*P* = 0.008; Fig. 1C) and T-score (*P* = 0.008) vs. the AMP group.

Based on reference whole-body BMD values from NHANES (Kelly, Wilson & Heymsfield, 2009), the mean value (± standard deviation) of T-score for the SCI and AMP groups was −1.25 (± 0.85) and −0.29 (±0.69), respectively. As shown in Fig. 2B, osteopenia and osteoporosis were present in 45.8% and 12.5% of athletes with SCI, respectively.

#### Regional analysis

One-way ANOVA showed that the %FM was significantly different between the SCI, AMP, and AB groups in all regions, namely the trunk (F = 20.027, *P* < 0.001, η^2^ = 0.331), android (F = 22.599, *P* < 0.001, η^2^ = 0.358), gynoid (F = 43.808, *P* < 0.001, η^2^ = 0.152), arms (F = 6.065, *P* = 0.004, η^2^ = 0.130), left/impaired leg (F = 50.645, *P* < 0.001, η^2^ = 0.556) and the right/non-impaired leg (F = 52.915, *P* < 0.001, η^2^ = 0.566) regions (Fig. 2A).

Post-hoc analysis showed that both the SCI and AMP groups had significantly higher %FM in the trunk (*P* < 0.001 and *P* = 0.012, respectively), android (*P* < 0.001 and *P* = 0.041, respectively), gynoid (*P* < 0.001 and *P* = 0.001, respectively), left/impaired leg (*P* < 0.001 for both) and right/non-impaired leg (*P* < 0.001 and *P* = 0.001, respectively) regions vs. the AB group (Fig. 1A). The SCI group had also significantly higher %FM in the arms vs. the AB group (*P* = 0.006; Fig. 1A), as well as higher %FM in the trunk (*P* = 0.014), android (*P* = 0.005), gynoid (*P* < 0.001) and right/non-affected leg (*P* < 0.001) regions in comparison with the AMP group (Fig. 1A).

**Figure 3:**
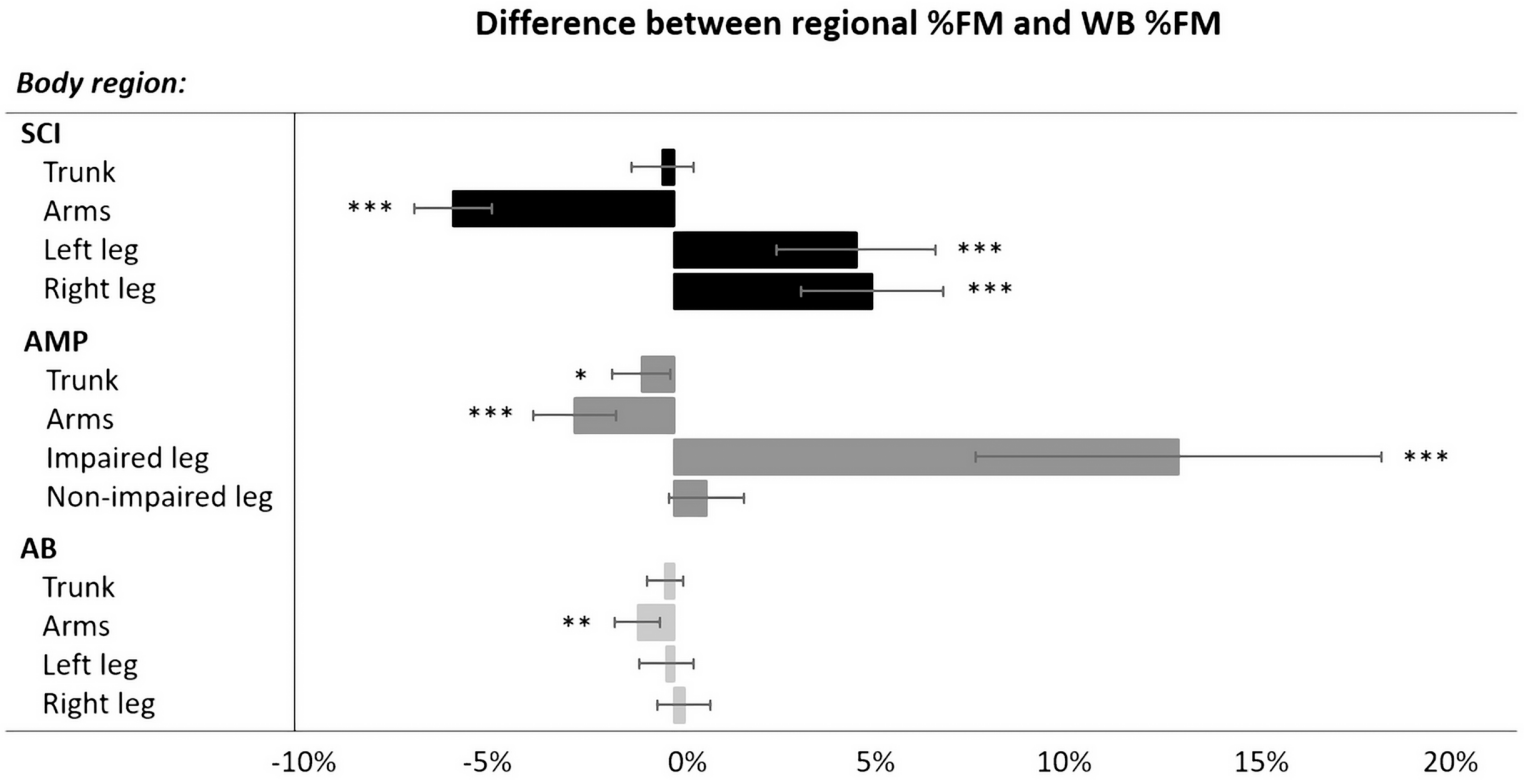
Differences (mean with Confidence Intervals) between regional %FM and whole-body %FM in each group (SCI, AMP and AB). SCI, spinal cord injury; AMP, unilateral lower limb amputation; AB, able-bodied; %FM, percentage fat mass; WB, whole-body. *P < 0.05; **P < 0.01; ***P < 0.001 (Paired-Samples T test, two-tailed).

Paired sample *t*-test showed that in the SCI group the %FM in the arms was significantly lower than the whole-body %FM (t = −11.10, *P* < 0.001, d = 0.93), while the %FM in both the left and right legs was statistically significant higher than that assessed at the whole-body level (t = 4.47, *P* < 0.001, d = 0.65 and t = 5.43, *P* < 0.001, d = 0.73, respectively) (Fig. 3). Similarly, in the AMP group the %FM in the arms was significantly lower than whole-body %FM (t = −4.78, *P* < 0.001, d = 0.59), while the %FM assessed in the trunk and in the impaired-leg only was significantly higher than whole-body %FM (t = −2.22, *P* = 0.040, d = 0.18 and t = 4.87, *P* < 0.001, d = 1.51, respectively) (Fig. 3). On the other hand, in the AB group only the %FM in the arms was significantly lower than the %FM assessed at the whole-body level (t = −3.27, *P* = 0.002, d = 0.31) (Fig. 3).

One-way ANOVA showed statistically significant differences between the SCI, AMP and AB groups for the FM/LM ratio in the trunk (F = 24.338, *P* < 0.001, η^2^ = 0.467), arm (F = 6.431, *P* = 0.003, η^2^ = 0.137) and leg regions (left/impaired leg: F = 26.064, η^2^ = 0.392; right/non-impaired leg: F = 42.787, η^2^ = 0.514; *P* < 0.001 for both). Post-hoc analysis showed that in both the SCI and the AMP groups FM/LM was significantly higher in the trunk (*P* < 0.001 and P 0.019, respectively; Fig. 1B) and in the legs (left/impaired leg: *P* < 0.001 for both; right/non-impaired leg: *P* < 0.001 and *P* = 0.018, respectively; Fig. 1B) in comparison with the AB group. The SCI group had significantly higher FM/LM ratio in the arms vs. the AB group (*P* = 0.003; Fig. 1B) as well as higher FM/LM ratio in the trunk (*P* = 0.006; Fig. 1B) and in the right/non-impaired leg (*P* < 0.001; Fig. 1B) in comparison with the AMP group (Fig. 1B).

As far as BMD is concerned, one-way ANOVA revealed statistically significant differences between the SCI, AMP and AB groups only in the legs (left/impaired leg: F = 38.703, η^2^ = 0.492; right/non-impaired leg: F = 49.529, η^2^ = 0.550; *P* < 0.001 for both; Fig. 1C). Post-hoc analysis showed that BMD was significantly lower in the left/non-impaired leg in both the SCI and AMP groups vs. the AB group (*P* < 0.001 for both; Fig. 1C). BMD in the right/non-impaired leg was significantly lower in the SCI group vs. both the AMP and AB groups (*P* < 0.001 for both; Fig. 1C).

### Impact of the severity of the disability on whole-body and regional body composition and BMD in athletes with SCI and in athletes with AMP

#### Whole-body analysis

Descriptive statistics (mean and standard deviation) of the whole-body body composition and BMD variables in the sub-groups of athletes with SCI (TETRA and PARA) as well as AMP (AKA and BKA) are reported in Table 2 and Table 3, respectively. At the whole-body level, the Student *t*-test for independent samples showed that TETRA and PARA groups had similar values for all body composition outcomes, BMD and T-score, whereas the AKA group was significantly different from the BKA group in DXA-measured total body mass, absolute LM and absolute BMC (Table 3). As shown in Table 2 and Table 3, the AKA and BKA groups were similar in FM, %FM, FM/LM, BMD and T-score.

**Table 2:** Body composition and BMD variables of the SCI group and its two sub-groups (TETRA and PARA groups).

	SCI (*n* = 24)		TETRA (*n* = 12)		PARA (*n* = 12)		Two-tailed Student *t*-test for independent samples		
	**Mean**	**SD**	**Mean**	**SD**	**Mean**	**SD**	**t**	**P**	**η**^**2**^
**Whole-body**									
Total mass (g)	70,774.29	12,430.77	70,579.99	14,737.57	70,968.58	10,286.76	−0.075	0.941	<0.001
LM (g)	50,039.05	6,718.44	4,9817.00	8,509.84	50,261.10	4,674.76	−0.158	0.876	0.001
FM (g)	18,474.39	6,843.86	18,416.37	7,159.12	18,532.40	6,831.87	−0.041	0.968	<0.001
%FM (%)	25.45	5.99	25.47	5.46	25.42	6.72	0.019	0.985	<0.001
FM/LM	0.37	0.11	0.36	0.10	0.37	0.12	−0.021	0.984	<0.001
BMC (g)	2,246.29	386.35	2,329.96	402.97	2,162.63	366.67	1.064	0.299	0.049
BMD (g/cm^2^)	1.07	0.09	1.09	0.08	1.06	0.10	0.640	0.529	0.018
**Trunk**									
Total mass (g)	36,040.80	7,020.49	35,603.05	7,906.65	36,478.56	6,334.20	−0.299	0.767	0.004
LM (g)	26,010.17	3,399.68	25,680.60	4,215.32	26,339.73	2,482.03	−0.467	0.645	0.010
FM (g)	9,423.76	4,128.76	9,308.39	4,244.27	9,539.14	4,195.26	−0.134	0.895	0.001
%FM (%)	25.14	6.89	25.18	6.49	25.10	7.55	0.029	0.977	<0.001
FM/LM	0.35	0.13	0.35	0.12	0.36	0.14	−0.019	0.985	<0.001
BMC (g)	604.59	115.53	614.06	106.58	595.12	127.87	0.394	0.697	0.007
BMD (g/cm^2^)	0.85	0.12	0.83	0.08	0.86	0.15	−0.486	0.632	0.011
**Android**									
FM (g)	1,773.36	944.38	1,780.97	961.62	1,765.75	969.51	0.039	0.970	<0.001
%FM (%)	30.18	9.26	30.11	8.27	30.26	10.53	−0.039	0.969	<0.001
**Gynoid**									
FM (g)	2,738.69	944.28	2,817.91	1,021.69	2,659.46	898.25	0.403	0.690	0.007
%FM (%)	31.17	8.08	30.56	7.05	31.78	9.27	−0.364	0.719	0.006
**Arms**									
Total mass (g)	10,174.88	1,901.80	9,370.24	1,920.15	10,979.51	1,569.36	**−2.248**	**0.035**	**0.187**
LM (g)	7,669.45	1,223.73	6,994.10	1,270.21	8,344.81	722.92	**−3.201**	**0.004**	**0.318**
FM (g)	2,063.06	940.74	1,941.35	882.68	2,184.77	1,019.31	−0.625	0.538	0.017
%FM (%)	19.66	6.44	20.18	6.20	19.14	6.90	0.388	0.702	0.007
FM/LM	0.27	0.11	0.28	0.11	0.26	0.11	0.393	0.698	0.007
BMC (g)	427.78	69.60	418.12	78.25	437.43	61.66	−0.671	0.509	0.020
BMD (g/cm^2^)	0.83	0.07	0.83	0.06	0.84	0.07	−0.450	0.657	0.009
**Left leg**									
Total mass (g)	9,778.46	2,064.08	10,284.24	2,567.74	9,272.67	1,325.43	1.213	0.238	0.063
LM (g)	6,511.57	1,520.85	6,883.13	1,839.71	6,140.01	1,072.59	1.209	0.240	0.062
FM (g)	2,967.91	1,022.68	3,073.14	1,046.17	2,862.68	1,033.52	0.496	0.625	0.011
%FM (%)	30.18	8.41	29.82	7.26	30.54	9.74	−0.205	0.839	0.002
FM/LM	0.48	0.22	0.47	0.20	0.49	0.24	−0.267	0.792	0.003
BMC (g)	298.97	95.90	327.97	98.18	269.98	88.07	1.523	0.142	0.095
BMD (g/cm^2^)	0.95	0.13	0.99	0.12	0.91	0.12	1.471	0.155	0.090
**Right leg**									
Total mass (g)	9,850.88	2,143.79	10,346.30	2,663.32	9,355.46	1,407.36	1.139	0.267	0.056
LM (g)	6,503.10	1,476.17	6,897.90	1,769.80	6,108.30	1,041.15	1.332	0.196	0.075
FM (g)	3,041.81	1,072.76	3,111.77	1,190.58	2,971.85	988.99	0.313	0.757	0.004
%FM (%)	30.59	8.03	29.74	7.45	31.43	8.82	−0.506	0.618	0.011
FM/LM	0.48	0.19	0.46	0.18	0.50	0.21	−0.506	0.618	0.012
BMC (g)	305.97	91.55	336.62	100.94	275.31	72.69	1.707	0.102	0.117
BMD (g/cm^2^)	0.97	0.12	1.00	0.13	0.94	0.11	1.334	0.196	0.075

**Note:**

SCI, athletes with spinal cord injury; TETRA, athletes with spinal cord injury at the cervical level; PARA, athletes with spinal cord injury at the thoracic/lumbar level; SD, standard deviation; η^2^, eta squared; LM, lean mass; FM, fat mass; %FM, percentage fat mass; FM/LM, fat-to-lean mass ratio; BMC, bone mineral content; BMD, bone mineral density. Statistically significant differences are in bold.

**Table 3:** Body composition and BMD variables of the AMP group and its two sub-groups (AKA and BKA groups).

	AMP (*n* = 18)		AKA (*n* = 11)		BKA (*n* = 7)		Two-tailed student *t*-test for independent samples		
	Mean	SD	Mean	SD	Mean	SD	t	*P*	η^2^
**Whole-body**									
Total mass (g)	67,823.84	12,138.98	62,114.11	8,638.53	76,796.26	11,808.54	**−3.053**	**0.008**	**0.368**
LM (g)	50,764.34	8,560.51	46,449.80	5,617.43	57,544.33	8,202.63	**−3.422**	**0.003**	**0.423**
FM (g)	14,737.93	4,342.30	13,523.11	4,281.06	16,646.93	3,990.12	−1.548	0.141	0.130
%FM (%)	21.45	4.21	21.45	5.06	21.47	2.76	−0.010	0.992	<0.001
FM/LM ratio	0.29	0.07	0.29	0.08	0.29	0.05	0.093	0.927	0.001
BMC (g)	2,321.57	328.17	2,141.21	260.64	2,605.00	196.36	**−4.021**	**0.001**	**0.503**
BMD (g/cm^2^)	1.17	0.07	1.15	0.07	1.20	0.08	−1.367	0.191	0.105
**Trunk**									
Total mass (g)	34,630.22	6,615.28	32,106.32	5,633.42	38,596.35	6,408.54	**−2.261**	**0.038**	**0.242**
LM (g)	26,716.82	4,639.99	24,737.51	3,833.42	29,827.17	4,247.26	**−2.636**	**0.018**	**0.303**
FM (g)	7,278.44	2,581.60	6,777.08	2,585.67	8,066.29	2,560.34	−1.035	0.316	0.063
%FM (%)	20.59	5.15	20.66	5.81	20.49	4.33	0.066	0.948	<0.001
FM/LM ratio	0.27	0.08	0.27	0.09	0.27	0.07	0.140	0.891	0.001
BMC (g)	634.96	102.04	591.74	97.96	702.89	68.56	**−2.610**	**0.019**	**0.299**
BMD (g/cm^2^)	0.88	0.08	0.87	0.09	0.91	0.06	−0.935	0.364	0.052
**Android**									
FM (g)	1,250.90	526.69	1,140.77	500.26	1,423.97	558.30	−1.120	0.279	0.073
%FM (%)	23.52	6.40	22.83	6.80	24.60	6.05	−0.563	0.581	0.019
**Gynoid**									
FM (g)	2,565.61	780.58	24,68.23	877.00	2,718.63	632.27	−0.652	0.524	0.026
%FM (%)	24.19	5.50	25.26	6.67	22.50	2.48	1.040	0.314	0.063
**Arms**									
Total mass (g)	9,687.03	1,861.72	9,223.93	1,551.03	10,414.75	2,190.84	−1.355	0.194	0.103
LM (g)	7,425.02	1,364.04	7,071.09	1,105.92	7,981.18	1,625.66	−1.421	0.175	0.112
FM (g)	1,853.65	621.59	1,764.10	665.66	1,994.37	564.27	−0.757	0.460	0.035
%FM (%)	18.84	4.63	18.77	5.62	18.94	2.85	−0.073	0.943	<0.001
FM/LM ratio	0.25	0.08	0.25	0.10	0.25	0.04	0.054	0.957	<0.001
BMC (g)	408.36	74.24	388.74	69.16	439.19	76.35	−1.451	0.166	0.116
BMD (g/cm^2^)	0.83	0.08	0.81	0.08	0.86	0.07	−1.309	0.209	0.097
**Impaired leg**									
Total mass (g)	5,465.89	3,095.04	3,330.28	1,183.59	8,821.84	1,818.10	**−7.810**	**<0.001**	**0.792**
LM (g)	3,625.51	2,296.23	2,050.89	886.40	6,099.93	1,393.16	**−7.585**	**<0.001**	**0.782**
FM (g)	1,693.45	803.98	1,198.01	513.46	2,472.01	484.59	**−5.240**	**<0.001**	**0.632**
%FM (%)	34.60	11.59	38.64	13.18	28.25	3.75	2.013	0.061	0.202
FM/LM ratio	0.61	0.39	0.74	0.46	0.41	0.08	1.829	0.086	0.173
BMC (g)	140.78	107.13	64.39	38.67	249.91	68.87	**−7.121**	**<0.001**	**0.760**
BMD (g/cm^2^)	1.04	0.15	1.00	0.16	1.11	0.12	−1.646	0.121	0.145
**Non-impaired leg**									
Total mass (g)	13,212.54	1,554.15	12,756.70	1,217.79	13,928.87	1,841.81	−1.635	0.122	0.143
LM (g)	9,728.93	1,085.28	9,395.31	877.02	10,253.18	1,237.15	−1.728	0.103	0.157
FM (g)	,2969.68	751.18	2,865.18	831.44	3,133.90	628.61	−0.730	0.476	0.032
%FM (%)	22.28	4.42	22.23	5.50	22.37	2.23	−0.065	0.949	<0.001
FM/LM ratio	0.31	0.08	0.31	0.10	0.30	0.04	0.072	0.944	<0.001
BMC (g)	513.94	60.72	496.21	51.34	541.79	67.61	−1.626	0.123	0.142
BMD (g/cm^2^)	1.27	0.12	1.25	0.09	1.30	0.15	−0.777	0.449	0.036

**Note:**

AMP, athletes with unilateral lower limb amputation; AKA, athletes with unilateral lower limb amputation above the knee; BKA, athletes with unilateral lower limb amputation below (or through) the knee; SD, standard deviation; η^2^, eta squared; LM, lean mass; FM, fat mass; %FM, percentage fat mass; FM/LM, fat-to-lean mass ratio; BMC, bone mineral content; BMD, bone mineral density. Statistically significant differences are in bold.

Based on %FM threshold of 25%, the highest frequency of obesity was found in the TETRA group (58.3%) (Fig. 1A). No athlete in the BKA group was obese, while 72.7% of athletes in the AKA group were (Fig. 1A).

According to reference whole-body BMD values from NHANES (Kelly, Wilson & Heymsfield, 2009), in the SCI group osteopenia was present in 50% of athletes in the TETRA group and 41.7% in the PARA group (Fig. 1B). As depicted in Fig. 1B, osteoporosis was present in one athlete in the TETRA group and in two athletes in the PARA group. In the two sub-groups of athletes with amputation, osteopenia was only present in the AKA group (4 athletes), while osteoporosis was absent in both the AKA and BKA groups (Fig. 1B).

#### Regional analysis

Descriptive statistic (mean and standard deviation) of the regional body composition and BMD variables in the sub-groups of athletes with SCI (TETRA and PARA groups) as well as in the sub-groups of athletes with AMP (AKA and BKA groups) are reported in Table 2 and Table 3, respectively. No statistically significant difference was found between TETRA and PARA but for the total mass and the absolute LM in the arms. In fact, the TETRA group had significantly lower total mass and LM in comparison with the PARA group (Table 2). When comparing the AKA and BKA groups, statistically significant differences were found for the total mass, LM and BMC in the trunk and in the impaired leg regions as well as in the FM of the impaired leg (Table 3).

Descriptive statistics (mean and standard deviation) of body composition and BMD assessed in the thigh and lower leg regions of both the impaired and non-impaired legs of athletes with amputation are reported in Table 4. In the impaired thigh, statistically significant lower total mass, LM, FM and BMC along with higher %FM was found in the AKA group vs. the BKA group (Table 4). Moreover, the AKA group had statistically significant lower LM and BMC in the non-impaired thigh and lower LM in the non-impaired lower leg versus the BKA group (Table 4).

**Table 4:** Segmental body composition and BMD variables of the AKA and BKA groups.

	AKA		BKA		Two-tailed student *t*-test for independent samples			
	Mean	SD	Mean	SD	t	*P*	η_p_^2^	g
**Impaired thigh**								
%FM (%)	34.58	9.98	26.50	4.19	2.007	0.063	0.212	0.948
FM/LM ratio	0.57	0.20	0.38	0.08	**2.438**	**0.028**	**0.284**	**1.120**
BMD (g/cm^2^)	1.00	0.29	1.16	0.16	−1.381	0.188	0.113	−0.613
**Non-impaired thigh**								
%FM (%)	21.72	5.64	20.66	3.12	0.453	0.657	0.013	0.209
FM/LM ratio	0.29	0.09	0.27	0.05	0.638	0.532	0.025	0.247
BMD (g/cm^2^)	1.44	0.10	1.57	0.16	−2.025	0.060	0.204	−0.875
**Non-impaired lower leg**								
%FM (%)	22.49	7.45	24.23	4.35	−0.556	0.586	0.019	−0.256
FM/LM ratio	0.33	0.14	0.35	0.09	−0.364	0.720	0.008	−0.153
BMD (g/cm^2^)	1.17	0.26	1.15	0.18	0.187	0.854	0.002	0.080

**Note:**

AKA, athletes with unilateral lower limb amputation above the knee; BKA, athletes with unilateral lower limb amputation below (or through) the knee; SD, standard deviation; g, Hedges’g; %FM, percentage fat mass; FM/LM, fat-to-lean mass ratio; BMD, bone mineral density. Statistically significant differences are in bold.

## Discussion

This study is the first investigation of the degree of alteration in DXA-measured whole-body and regional body composition and BMD in athletes with a physical impairment according to the type of disability (i.e., SCI vs. AMP) and the degree of severity (i.e., TETRA vs. PARA and AKA vs. BKA).

In summary, the results demonstrated the following points:

1. Whole-body body composition is altered in both the SCI and AMP groups vs. the AB group, but the extent of such changes is more severe in athletes with SCI.
2. Whole-body BMD was only affected in athletes with SCI, with about half of them showing osteopenia or osteoporosis.
3. Regionally, the type of physical impairment influenced the extent of body composition alterations, which was more serious in athletes with SCI. The regional %FM and BMD were impairment specific.
4. The severity of physical impairment affected regional body composition in the arms of athletes with SCI and the impaired and non-impaired legs of athletes with AMP.

### Impact of the severity of the disability on whole-body and regional body composition and BMD in athletes with a physical impairment

#### Whole-body

A first result of the present study was that athletes with a physical impairment have a higher accrual of FM and/or a reduction in LM leading to a statistically significant increase in %FM and FM/LM ratio in comparison with AB athletes irrespective of the type of impairment. Bearing in mind that the three groups of male athletes were similar in age, this result suggests that both types of physical impairment (SCI, AMP) can produce whole-body alterations in body composition. This result was expected and in line with previous findings in this athletic population (Cavedon, Zancanaro & Milanese, 2020; Miyahara et al., 2008). Interestingly, the alterations in whole-body body composition were more severe in athletes with SCI. In fact, athletes with SCI had significantly higher %FM (+4.0%) and FM/LM ratio (+21.6%) vs. AMP athletes. Moreover, most athletes with SCI (58.3%) had a %FM equal to or greater than 25% and therefore are considered obese (Romero-Corral et al., 2008); the prevalence of obesity was obviously lower in the group of athletes with AMP (16.7%). Since the SCI and AMP groups were similar for age, DOI, sport experience and weekly amount of training, it is reasonable to assume that the extent of alterations in body composition in this athletic population may be impairment specific. This can be explained by the fact that a paralysis due to SCI would affect more parts of the body than the amputation of a lower limb, and would prevent the possibility of movement in part of the trunk and in the legs, reducing the resting metabolic rate and physical activity levels. Accordingly, it can be argued that SCI would induce a greater imbalance between energy expenditure and energy intake when compared to AMP leading to more serious alterations in whole-body body composition. As a consequence, athletes with SCI may be more at risk of developing the well-known negative health consequences of excess body fat when compared to athletes with AMP (Dionyssiotis, 2019; Gater et al., 2019). This result advocates that strategies (e.g., nutrition programs, training plans, healthy lifestyle guidelines) aimed at improving body composition in athletes with a physical impairment are of great importance in this athletic population and should be specific for the type of physical impairment (SCI or AMP).

Another interesting result of the present study was that SCI had a negative impact on whole-body bone health of athletes with a physical impairment, whereas AMP did not. In fact, athletes with SCI had significantly lower BMD and T-score values versus both the AMP and AB groups, while athletes with AMP had similar values of BMD and T-score in comparison with AB athletes. In addition, as reported in Fig. 2, more than half of athletes in the SCI group (56.0%) were osteopenic (11 out of 25) or osteoporotic (3 out of 25), while a limited proportion of athletes with AMP suffered from osteopenia or osteoporosis (4 out of 18). This result can be attributed to the fact that athletes with SCI were all wheelchair-dependent for daily living whereas athletes with AMP were all able to walk even if with the aid of prosthetics or crutches. During human locomotion, most of the mechanical forces that act on the skeleton are generated either through impact with the ground (i.e., gravitational or ground-reaction forces) or through skeletal muscle contractions (i.e., muscle or joint-reaction forces). The decline of locomotion abilities in athletes with SCI, who are seated for most of the time, leads to mobility limitations or even immobility, especially in the lower body, and the mechanical unloading associated with prolonged immobility is typically associated with bone resorption (Ashe et al., 2006; Lam & Qin, 2008). What is more, these results suggest the importance of dosing Vitamin D and Calcium in athletes with SCI thereby recommending appropriate nutritional supplementations in case of deficiency of these micronutrients.

#### Regional analysis

The results of the present study showed that body composition at the regional level was altered in both the SCI and AMP groups vs. the AB group. In fact, in comparison with AB athletes, both groups of athletes with a physical impairment had significantly higher %FM and FM/LM in all regions with the exceptions of the arms. The absence of body composition alterations in the arms in both the SCI and AMP groups was expected. Lower values of %FM found in the arms of athletes with SCI were in line with the literature (Inukai et al., 2006; Miyahara et al., 2008) and can be explained by the fact that the arms are greatly employed by athletes with SCI both in activities of daily life (e.g., pushing the hand-propelled wheelchair) as well as in activities related to the sport practiced (e.g., pushing the sport-specific wheelchair, dribbling and passing the ball). Moreover, even if athletes with AMP were able to walk during daily life, they employed their arms extensively during sport practice by using their crutches (e.g., in the case of amputee soccer players) or to propel their wheelchair during sport activities (e.g. in the case of wheelchair basketball players).

An intriguing finding of this study was that the %FM changes with the body regions in athletes with a physical impairment while in AB athletes the %FM is similar in all regions. More specifically, in AB athletes the %FM in the arms (15.7%), legs (16.7%) and trunk (16.4%) regions were close to the whole-body %FM value (16.7%). Instead, the SCI group had significantly lower (−5.8%) %FM in the arms along with significantly higher (+4.94%) %FM in the legs in comparison with the whole-body %FM value. This trend was in line with that reported in previous investigations (Inukai et al., 2006; Miyahara et al., 2008) showing that in athletes with SCI the %FM varies across the body regions being greater in the sub-lesional regions in comparison with the upper body regions. Instead, the regional %FM in athletes with AMP was characterized by a significantly lower (−2.6%) value in the arms vs. the whole-body %FM and by an asymmetrical %FM in the legs with the %FM in the impaired leg being significantly higher than that at the whole-body level (+13.5%). The results of the present study on athletes with AMP expand on previous investigations on non-athletic populations with AMP (Sherk, Bemben & Bemben, 2008; Bemben et al., 2017), which reported that the regional alterations in body composition mainly affected the impaired limb and involved muscle atrophy and an increase in the amount of FM.

The current results provide an explanation for the low reliability of anthropometric equations validated in healthy populations in predicting the DXA-measured %FM in athletes with a physical impairment (Mojtahedi, Valentine & Evans, 2009; Willems et al., 2015; Goosey-Tolfrey et al., 2016). Authors assumed that this was due to the altered and/or asymmetrical regional distribution of FM in athletes with a physical impairment. By assessing the %FM at the regional level in athletes with SCI or AMP, our results offer a rationale for the lack of reliability between the %FM assessed through anthropometric equations and that assessed by means of DXA. Furthermore, the results of the present study provided evidence for the need to develop population-specific anthropometric equations to accurately predict body composition in this athletic population when DXA is not available.

The higher %FM in the android and gynoid regions in SCI vs. AMP athletes is of clinical significance showing that SCI athletes are at increased risk of metabolic syndrome and cardiovascular disease (Toresdahl et al., 2019), caused by an excess of central adiposity (Goldstein et al., 2017; Stillman & Williams, 2019).

This finding was in agreement with previous investigations dealing with non-athletic populations with SCI (Spungen et al., 2003; Gorgey & Gater, 2011). However, it is intriguing that athletes with SCI had lower %FM in the android and gynoid regions in comparison with that reported in the literature for non-athletic people with SCI (Goldstein et al., 2017; Gorgey et al., 2018). In fact, athletes with SCI in the present study had a %FM of about 30% in the android and gynoid regions while Goldstein and colleagues (2017) and Gorgey and colleagues (2018) reported values close to 40%. As a consequence, it is suggested that sport practice may help to mitigate the adverse changes in body composition in this population and support the importance of studying the impact of such modifiable factors on the health of people with SCI.

Taken together these results underlined that the regional alterations in body composition are impairment-specific and therefore training protocols which take the athlete’s type of impairment into account should be encouraged in this population, especially from a health perspective.

As far as regional BMD is concerned, the current results showed that the type of physical impairment affects bone health at the regional level. In fact, athletes with SCI had reduced BMD in both legs whereas athletes with AMP had reduced BMD in the impaired lower leg only (Fig. 1C). Lower BMD in the legs of wheelchair athletes was also found in the study conducted by Miyahara and colleagues (2008) and can be explained by the serious adverse effects of immobilization on leg bones associated with a reduction of the effects of gravity and denervation atrophy (Spungen et al., 2003; Mojtahedi, Valentine & Evans, 2009). Our findings expand on previous findings on non-athletic subjects with AMP (Sherk, Bemben & Bemben, 2008; Bemben et al., 2017) showing that bone demineralization in the impaired leg also occurs in athletes with AMP despite the practice of sport activity. In the future it would be interesting to explore whether the type of locomotion used in the sport practiced by athletes with AMP may have an impact on BMD in the impaired leg (e.g., wheelchair sports, prosthetic sports like track and field and Para Table Tennis, unassisted sports like Para Swimming).

### The impact of the severity of the disability on whole-body and regional body composition and BMD in athletes with SCI and in athletes with AMP

In athletes with SCI the severity of disability (i.e., SCI at the cervical level [TETRA] versus SCI at the thoracic/lumbar level [PARA]) did not have an impact on whole-body body composition and BMD. This finding is supported by previous findings in athletes with SCI (Inukai et al., 2006; Goosey-Tolfrey et al., 2016; Flueck, 2020), where no significant differences in whole-body body composition in athletes with SCI with respect to the level of the lesion was reported. In this study, no significant differences were found at the regional level either, with the exception of the arms where the severity of disability has been shown to affect the total mass and the LM of athletes with SCI. In particular, the TETRA group had significantly lower total mass and LM in the arms in comparison with the PARA group by respectively 17.2% and 19.3%. This finding was in contrast with a recent investigation on athletes with SCI (Flueck, 2020) reporting that the regional differences in body composition between athletes with tetraplegia and athletes with paraplegia occurred in the legs. Furthermore, two studies (Singh et al., 2014; Gorgey et al., 2015) on non-athletic people with SCI also reported that that the sub-lesional differences in body composition depend on the level of the injury. Due to the paucity of studies investigating the impact of the severity of disability of athletes with SCI, this point remains controversial and requires further and more broadened investigations.

In athletes with AMP the results showed that the severity of the physical impairment (i.e., above-knee amputation [AKA] or below-knee amputation [BKA]) has an impact on whole-body body composition resulting in lower total mass, LM and BMC in AKA vs. BKA. This result was expected, and it is obviously due to the absence of a larger part of the body in AKA. Due to the absence of scientific information on body composition in athletes with AMP, we thought it useful to enrich the scientific literature in this field of research with an accurate quantification of such a difference. It is interesting to underline that when considering the variables of body composition expressed in relative terms (i.e., %FM, FM/LM and BMD) no significant differences were found between AKA and BKA. This result implies that in athletes with AMP the severity of their impairment does not seem to have an effect on the accumulation of FM associated with a reduction of LM or on BMC at the whole-body level. At the regional level, in the trunk region of athletes with AMP, the results showed a trend similar to that previously reported at the whole-body level. This trend may be due to the fact that the trunk region, as measured by DXA, is a standard region of interest which also includes the pelvic triangle. In fact, the two lower boundaries of the trunk region are represented by two oblique lines that divide the neck of the femur in half. Accordingly, the presence of athletes in the AKA group with amelia or with an amputation through the hip (i.e., athletes where the head and neck of the femur are missing) can reasonably be considered the reason for such differences in the trunk region of this group, rather than it being a true difference between the two groups due to the severity of the physical impairment.

Of interest, in line with the result reported at the whole-body level, the severity of the AMP did not impact the regional body composition in athletes with AMP in the android, gynoid and arms regions. As expected, in the impaired thigh of athletes with AMP the severity of the disability affects the total mass and all its components (i.e., LM, FM and BMC) which are lower in athletes with AKA by respectively 164.9%, 197.4%, 106.3% and 288.1% versus athletes with BKA. These results were in agreement with previous findings (Sherk, Bemben & Bemben, 2008, 2010) on non-athletic people with AMP reporting that in people with unilateral lower limb amputation, body composition is influenced by the level of amputation, with above-knee amputees having greater FM and bone atrophy in the impaired limb than the below-knee amputees. Surprisingly, the severity of disability has an impact on body composition in the non-impaired leg of athletes with AMP. Comparing athletes with AKA vs. those with BKA, reduced LM and BMC in the thigh as well as reduced LM in the lower leg were found. In future research, it would be interesting to understand whether such differences in the non-impaired leg (both thigh and lower leg segments) are effectively due to the severity of the disability or if they are caused by other factors (e.g. the practice of a wheelchair sport versus a sport which requires the use of a prosthetic).

#### Limitations and strengths

This study has some limitations that should be mentioned. First, the relatively limited number of athletes with a physical impairment prevented the investigation of the combined effect of both the sport practiced and the type and the severity of the disability on whole-body and regional body composition. Second, information about the basal metabolism of the participants was not known. Third, information on the dietary habits of the subjects was not collected. Future research is therefore needed to consider the combined effect of both the type and severity of the physical impairment and the dietary habits of athletes with a physical impairment on their body composition and bone health.

In this work, however, there are also a number of strengths to be underlined. First, to the best of our knowledge this study is the first to report body composition and BMD variables as measured by DXA in AMP athletes considered as an independent group. Second, this study is the first to provide data on FM in the android and gynoid regions, whole-body and regional FM/LM ratio and T-score values for the definition of osteopenia or osteoporosis in athletes with SCI. Third, using DXA to assess body composition, both the whole-body and regional body composition of participants were reliably measured (Mojtahedi, Valentine & Evans, 2009; Sutton et al., 2009; Keil et al., 2016). Fourth, the control group was representative of a healthy athletic population and was carefully age-matched with athletes with a physical impairment.

#### Practical applications

The results of the present study underly the need for nutritionists, medical sports doctors, clinicians, physical conditioners and coaches to consider that the type of physical impairment has an impact on body composition and BMD as well as on the regional distribution of %FM in athletes with a disability. Accordingly, when considering issues on body composition and BMD, athletes with SCI or AMP should be considered separately. From a practical perspective, this would for example imply that nutritional interventions and training programs aimed at improving body composition and bone health in athletes with a physical impairment should be specific for the type of the disability. Moreover, physical conditioners and coaches dealing with body composition in athletes with AMP, should also consider strategies to improve body composition in the impaired lower limb according to the severity of the physical impairment of athletes.

Other important practical applications arising from the results of this study concern the methodologies adopted to estimate body composition in this athletic population. For instance, athletes with SCI or AMP should be considered separately when investigating the capability of some field-based methods (e.g., skinfold thickness technique and bioimpedentiometry) to estimate the DXA-measured body composition in athletes with a physical impairment. What is more, these results underpin the need for population-specific equations based upon the type of physical impairment in order to estimate body composition by-means of skinfold thickness, ultrasound or bioimpedentiometry in athletes with a physical impairment.

## Conclusions

In conclusion, providing accurate information on DXA-measured body composition and BMD in athletes with a physical impairment is important from health, nutrition, methodological and sport-related performance perspectives. The findings of the present study provide evidence that athletes with SCI and athletes with AMP have different body composition characteristics and different patterns of %FM distribution at the regional level. The results of this study help in filling some important gaps in the scientific literature by providing a better understanding of alterations in whole-body and regional body composition in athletes with a physical impairment. These results also open new perspectives on the assessment of body composition in athletes with a physical impairment by underlining the need to consider athletes with SCI or AMP separately.

## Supplemental Information

10.7717/peerj.11296/supp-1 Supplemental Information 1 DXA-measured raw data.

10.7717/peerj.11296/supp-2 Supplemental Information 2 Legend to interpret the DXA-measured raw data.

## Additional Information and Declarations

### Data Availability

The following information was supplied regarding data availability: The DXA measurements and legend to interpret the DXA measurements are available in the Supplemental Files.
